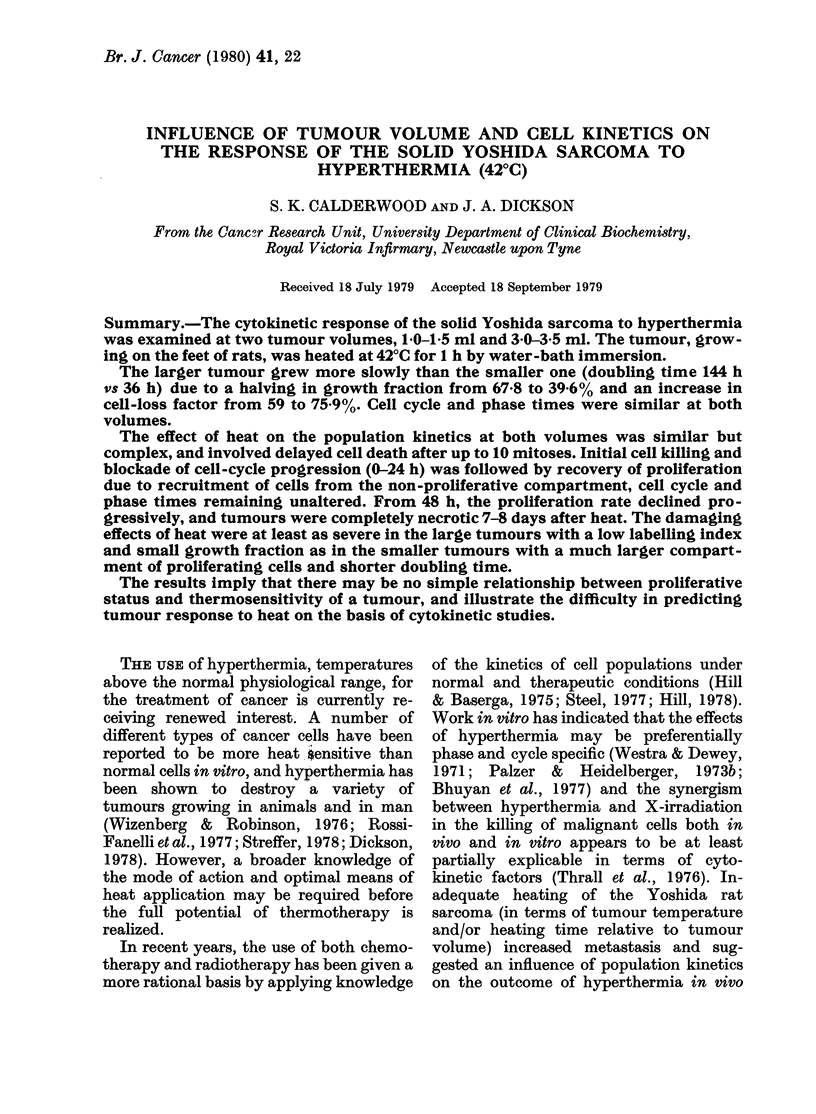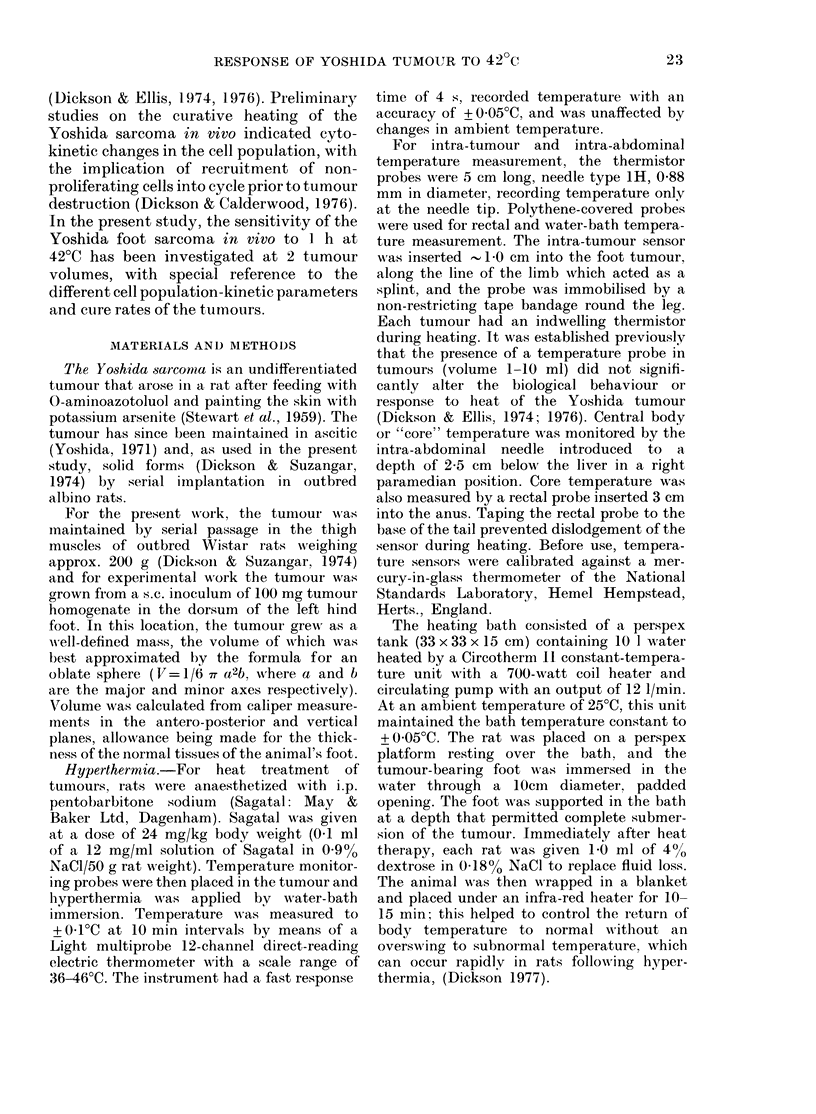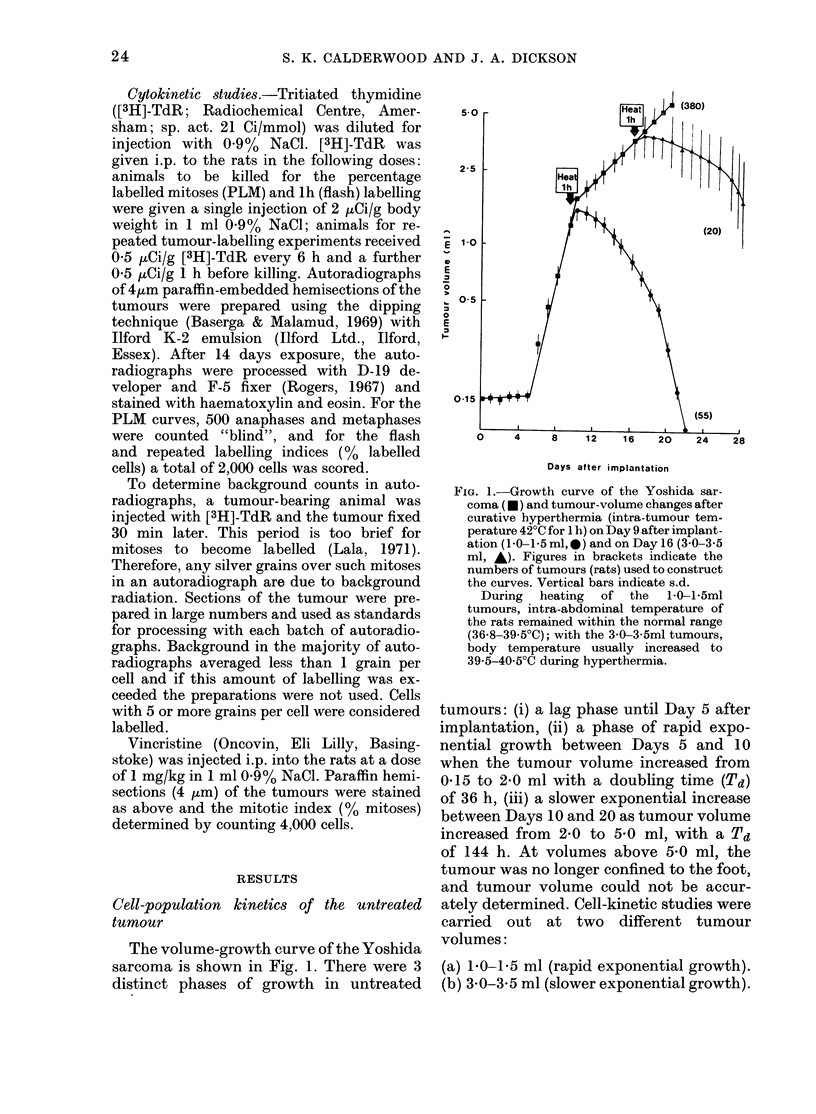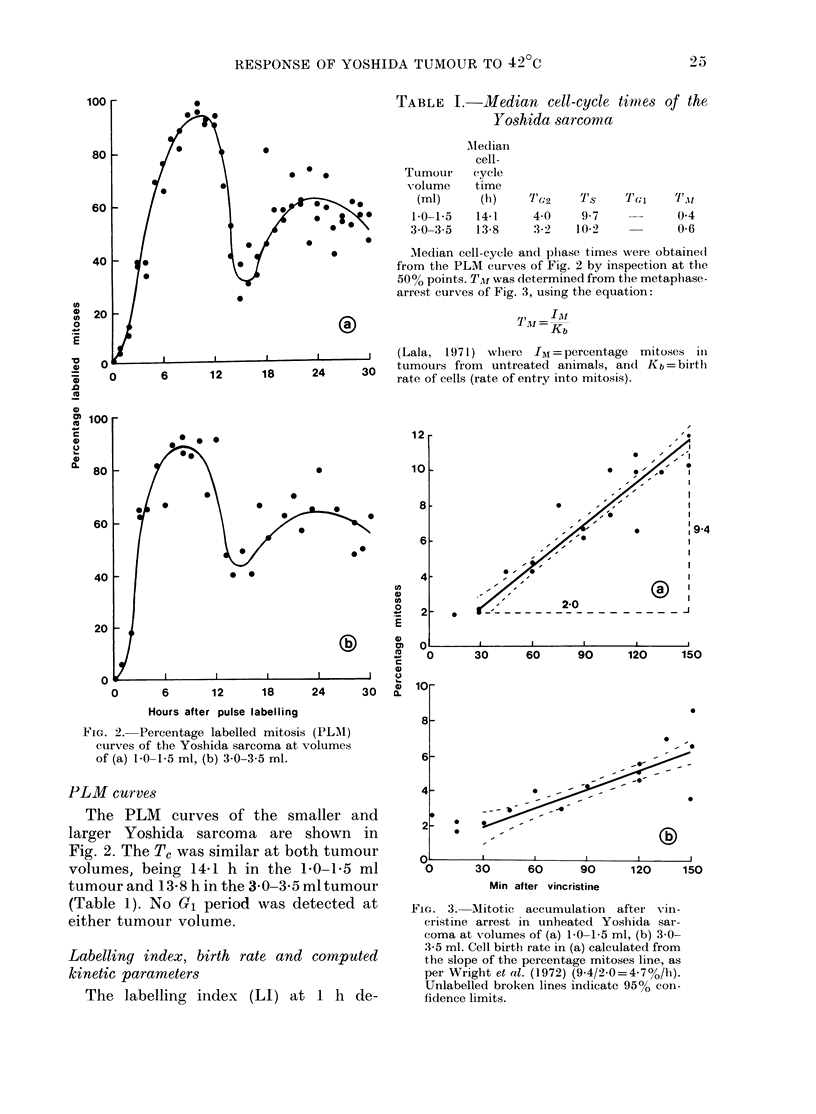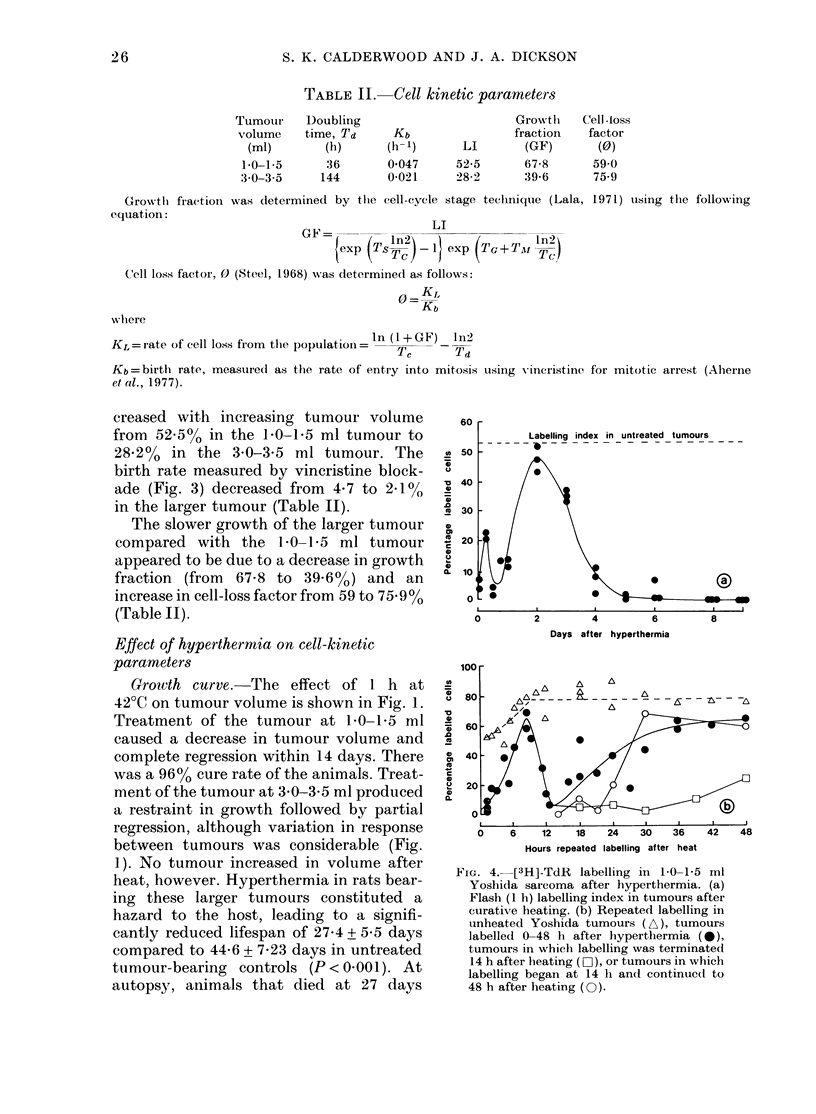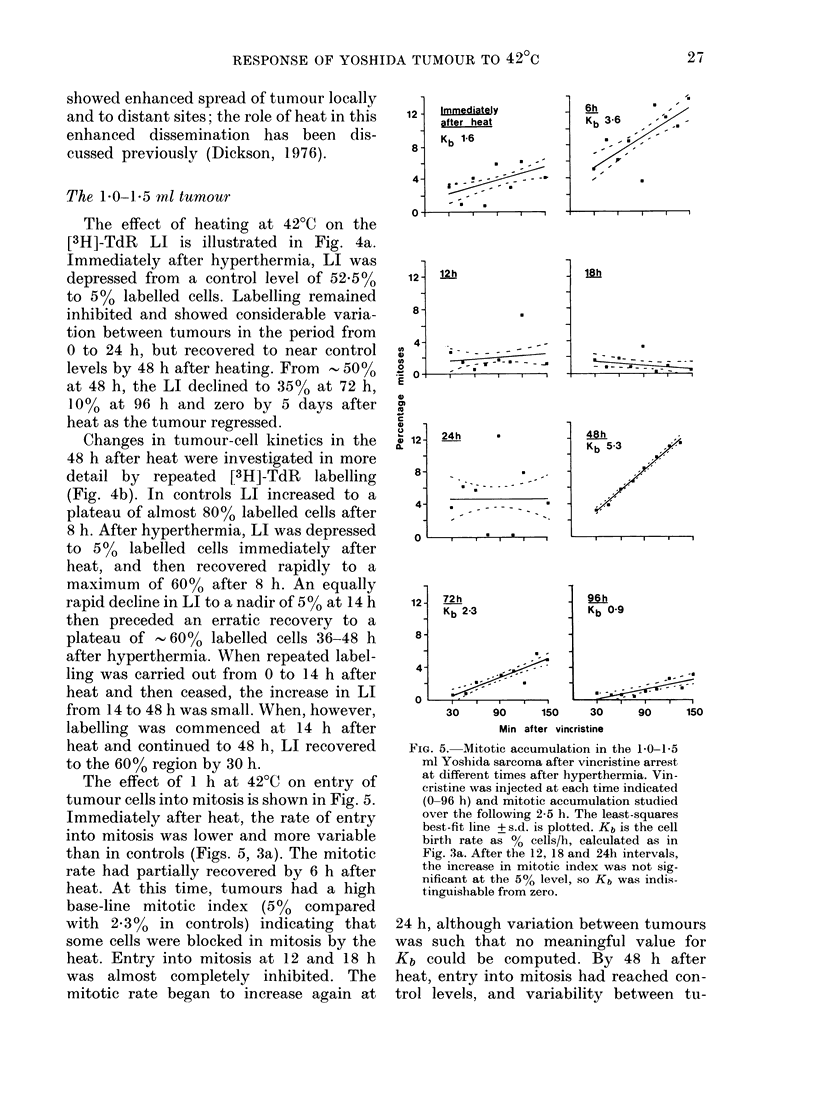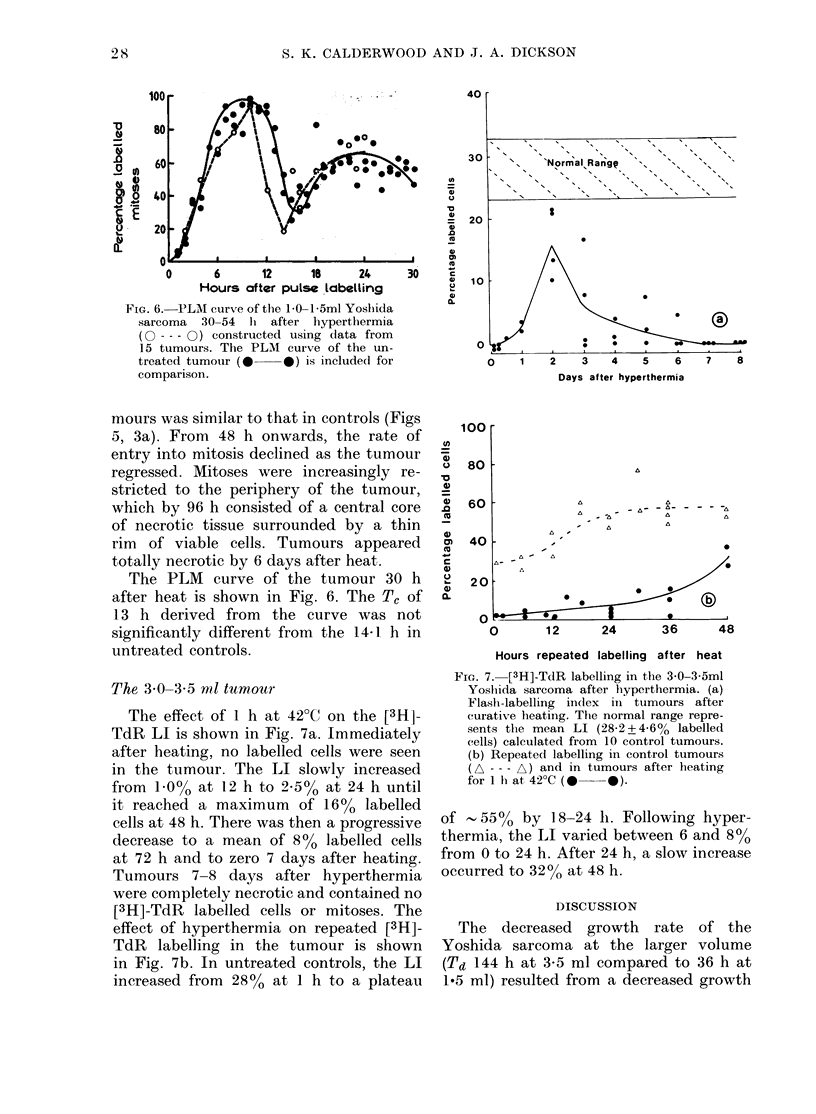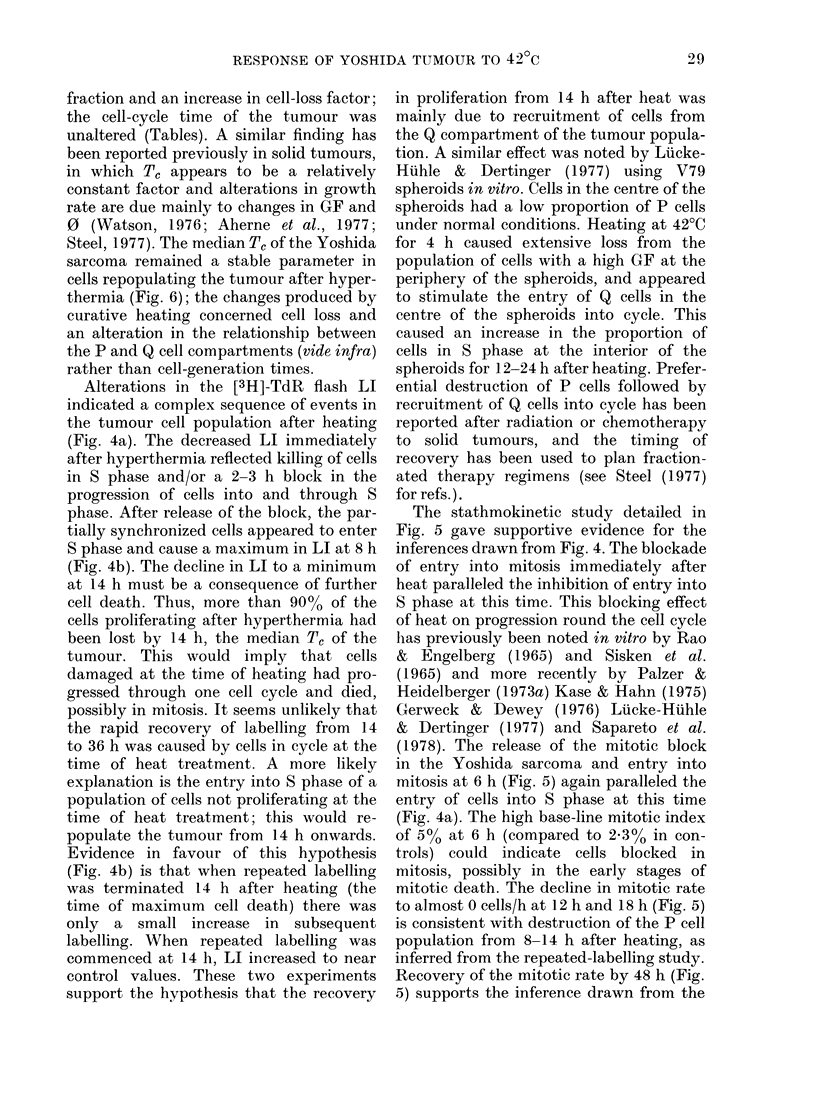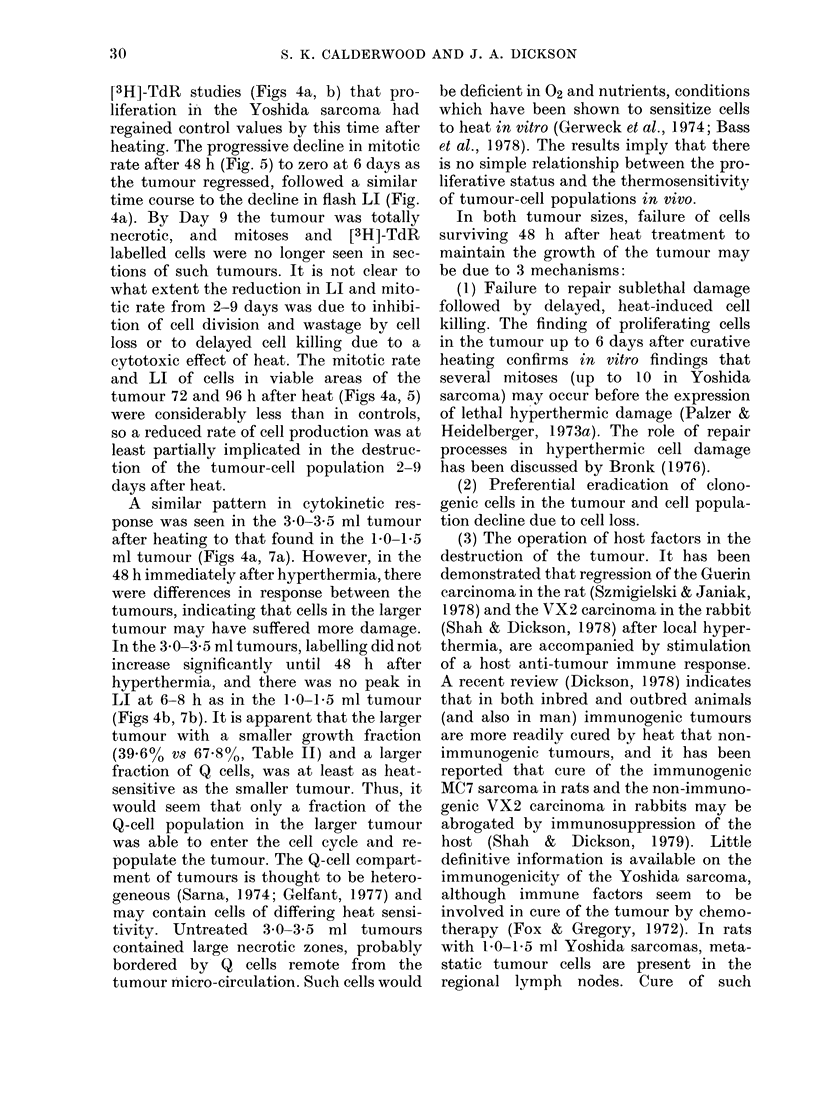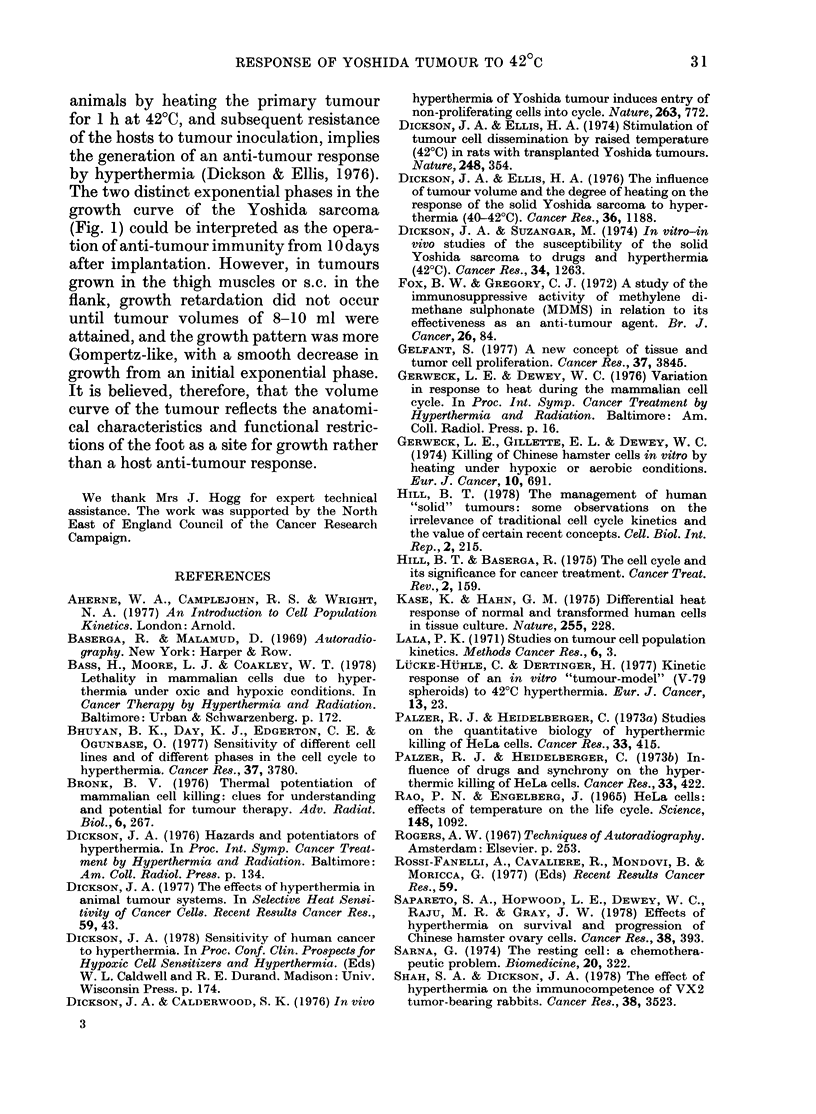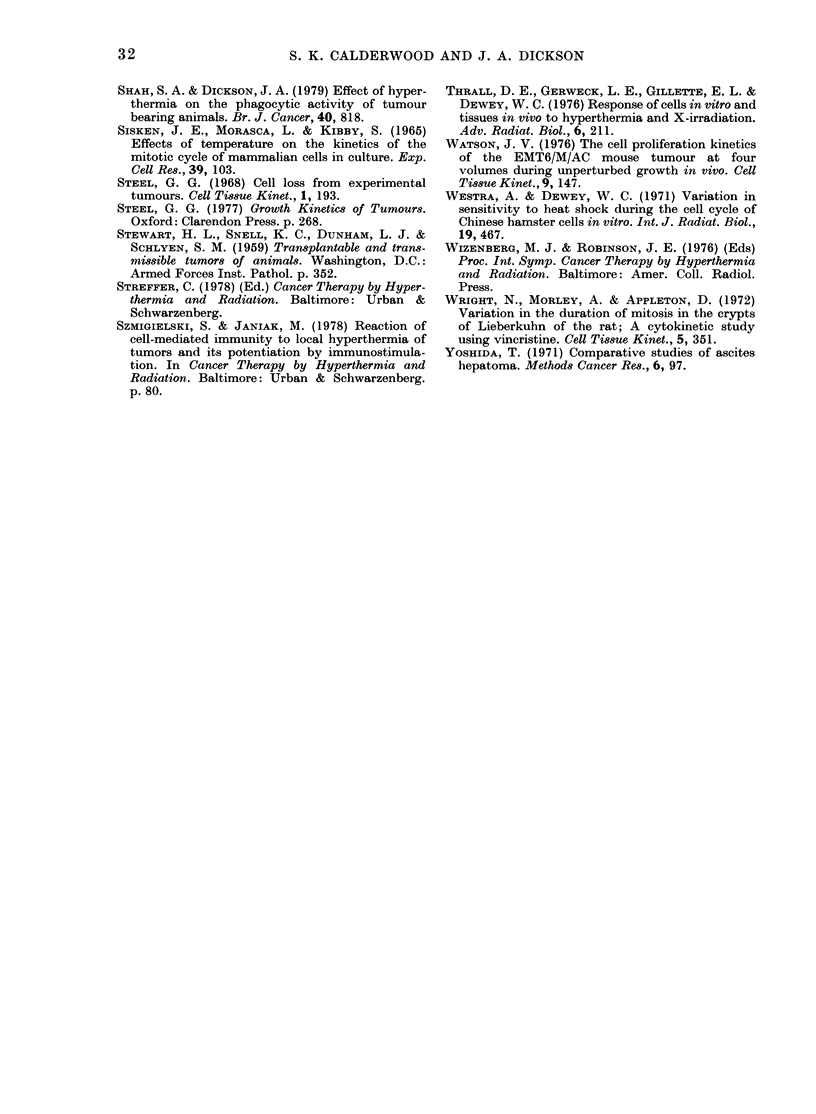# Influence of tumour volume and cell kinetics on the response of the solid Yoshida sarcoma to hyperthermia (42 degrees C).

**DOI:** 10.1038/bjc.1980.3

**Published:** 1980-01

**Authors:** S. K. Calderwood, J. A. Dickson

## Abstract

The cytokinetic response of the solid Yoshida sarcoma to hyperthermia was examined at two tumour volumes, 1.0-1.5 ml and 3.0-3.5 ml. The tumour, growing on the feet of rats, was heated at 42 degrees C for 1 h by water-bath immersion. The larger tumour grew more slowly than the smaller one (doubling time 144 h vs 36 h) due to a halving in growth fraction from 67.8 to 39.6% and an increase in cell-loss factor from 59 to 75.9%. Cell cycle and phase times were similar at both volumes. The effect of heat on the population kinetics at both volumes was similar but complex, and involved delayed cell death after up to 10 mitoses. Initial cell killing and blockade of cell-cycle progression (0-24 h) was followed by recovery of proliferation due to recruitment of cells from the non-proliferative compartment, cell cycle and phase times remaining unaltered. From 48 h, the proliferation rate declined progressively, and tumours were completely necrotic 7-8 days after heat. The damaging effects of heat were at least as severe in the large tumours with a low labelling index and small growth fraction as in the smaller tumours with a much larger compartment of proliferating cells and shorter doubling time. The results imply that there may be no simple relationship between proliferative status and thermosensitivity of a tumour, and illustrate the difficulty in predicting tumour response to heat on the basis of cytokinetic studies.


*Br. J. Cancer* (1980) **41**, 22

### INFLUENCE OF TUMOUR VOLUME AND CELL KINETICS ON THE RESPONSE OF THE SOLID YOSHIDA SARCOMA TO HYPERTHERMIA (42°C)

S. K. CALDERWOOD AND J. A. DICKSON

*From the Cancer Research Unit, University Department of Clinical Biochemistry, Royal Victoria Infirmary, Newcastle upon Tyne*

Received 18 July 1979 Accepted 18 September 1979

**Summary.—The cytokinetic response of the solid Yoshida sarcoma to hyperthermia was examined at two tumour volumes, 1·0–1·5 ml and 3·0–3·5 ml. The tumour, growing on the feet of rats, was heated at 42°C for 1 h by water-bath immersion.**

**The larger tumour grew more slowly than the smaller one (doubling time 144 h *vs* 36 h) due to a halving in growth fraction from 67·8 to 39·6% and an increase in cell-loss factor from 59 to 75·9%. Cell cycle and phase times were similar at both volumes.**

**The effect of heat on the population kinetics at both volumes was similar but complex, and involved delayed cell death after up to 10 mitoses. Initial cell killing and blockade of cell-cycle progression (0–24 h) was followed by recovery of proliferation due to recruitment of cells from the non-proliferative compartment, cell cycle and phase times remaining unaltered. From 48 h, the proliferation rate declined progressively, and tumours were completely necrotic 7–8 days after heat. The damaging effects of heat were at least as severe in the large tumours with a low labelling index and small growth fraction as in the smaller tumours with a much larger compartment of proliferating cells and shorter doubling time.**

**The results imply that there may be no simple relationship between proliferative status and thermosensitivity of a tumour, and illustrate the difficulty in predicting tumour response to heat on the basis of cytokinetic studies.**

THE USE of hyperthermia, temperatures above the normal physiological range, for the treatment of cancer is currently receiving renewed interest. A number of different types of cancer cells have been reported to be more heat sensitive than normal cells *in vitro*, and hyperthermia has been shown to destroy a variety of tumours growing in animals and in man (Wizenberg & Robinson, 1976; Rossi-Fanelli *et al.*, 1977; Streffer, 1978; Dickson, 1978). However, a broader knowledge of the mode of action and optimal means of heat application may be required before the full potential of thermotherapy is realized.

In recent years, the use of both chemotherapy and radiotherapy has been given a more rational basis by applying knowledge of the kinetics of cell populations under normal and therapeutic conditions (Hill & Baserga, 1975; Steel, 1977; Hill, 1978). Work *in vitro* has indicated that the effects of hyperthermia may be preferentially phase and cycle specific (Westra & Dewey, 1971; Palzer & Heidelberger, 1973*b*; Bhuyan *et al.*, 1977) and the synergism between hyperthermia and X-irradiation in the killing of malignant cells both *in vivo* and *in vitro* appears to be at least partially explicable in terms of cytokinetic factors (Thrall *et al.*, 1976). Inadequate heating of the Yoshida rat sarcoma (in terms of tumour temperature and/or heating time relative to tumour volume) increased metastasis and suggested an influence of population kinetics on the outcome of hyperthermia *in vivo*

(Dickson & Ellis, 1974, 1976). Preliminary studies on the curative heating of the Yoshida sarcoma *in vivo* indicated cytokinetic changes in the cell population, with the implication of recruitment of non-proliferating cells into cycle prior to tumour destruction (Dickson & Calderwood, 1976). In the present study, the sensitivity of the Yoshida foot sarcoma *in vivo* to 1 h at 42°C has been investigated at 2 tumour volumes, with special reference to the different cell population-kinetic parameters and cure rates of the tumours.

### MATERIALS AND METHODS

*The Yoshida sarcoma* is an undifferentiated tumour that arose in a rat after feeding with O-aminoazotoluol and painting the skin with potassium arsenite (Stewart *et al.*, 1959). The tumour has since been maintained in ascitic (Yoshida, 1971) and, as used in the present study, solid forms (Dickson & Suzangar, 1974) by serial implantation in outbred albino rats.

For the present work, the tumour was maintained by serial passage in the thigh muscles of outbred Wistar rats weighing approx. 200 g (Dickson & Suzangar, 1974) and for experimental work the tumour was grown from a s.c. inoculum of 100 mg tumour homogenate in the dorsum of the left hind foot. In this location, the tumour grew as a well-defined mass, the volume of which was best approximated by the formula for an oblate sphere ($V = 1/6\ \pi\ a^2b$, where $a$ and $b$ are the major and minor axes respectively). Volume was calculated from caliper measurements in the antero-posterior and vertical planes, allowance being made for the thickness of the normal tissues of the animal's foot.

*Hyperthermia.*—For heat treatment of tumours, rats were anaesthetized with i.p. pentobarbitone sodium (Sagatal: May & Baker Ltd, Dagenham). Sagatal was given at a dose of 24 mg/kg body weight (0·1 ml of a 12 mg/ml solution of Sagatal in 0·9% NaCl/50 g rat weight). Temperature monitoring probes were then placed in the tumour and hyperthermia was applied by water-bath immersion. Temperature was measured to ±0·1°C at 10 min intervals by means of a Light multiprobe 12-channel direct-reading electric thermometer with a scale range of 36–46°C. The instrument had a fast response time of 4 s, recorded temperature with an accuracy of ±0·05°C, and was unaffected by changes in ambient temperature.

For intra-tumour and intra-abdominal temperature measurement, the thermistor probes were 5 cm long, needle type 1H, 0·88 mm in diameter, recording temperature only at the needle tip. Polythene-covered probes were used for rectal and water-bath temperature measurement. The intra-tumour sensor was inserted ~1·0 cm into the foot tumour, along the line of the limb which acted as a splint, and the probe was immobilised by a non-restricting tape bandage round the leg. Each tumour had an indwelling thermistor during heating. It was established previously that the presence of a temperature probe in tumours (volume 1–10 ml) did not significantly alter the biological behaviour or response to heat of the Yoshida tumour (Dickson & Ellis, 1974; 1976). Central body or "core" temperature was monitored by the intra-abdominal needle introduced to a depth of 2·5 cm below the liver in a right paramedian position. Core temperature was also measured by a rectal probe inserted 3 cm into the anus. Taping the rectal probe to the base of the tail prevented dislodgement of the sensor during heating. Before use, temperature sensors were calibrated against a mercury-in-glass thermometer of the National Standards Laboratory, Hemel Hempstead, Herts., England.

The heating bath consisted of a perspex tank (33 × 33 × 15 cm) containing 10 l water heated by a Circotherm II constant-temperature unit with a 700-watt coil heater and circulating pump with an output of 12 l/min. At an ambient temperature of 25°C, this unit maintained the bath temperature constant to ±0·05°C. The rat was placed on a perspex platform resting over the bath, and the tumour-bearing foot was immersed in the water through a 10cm diameter, padded opening. The foot was supported in the bath at a depth that permitted complete submersion of the tumour. Immediately after heat therapy, each rat was given 1·0 ml of 4% dextrose in 0·18% NaCl to replace fluid loss. The animal was then wrapped in a blanket and placed under an infra-red heater for 10–15 min; this helped to control the return of body temperature to normal without an overswing to subnormal temperature, which can occur rapidly in rats following hyperthermia, (Dickson 1977).

*Cytokinetic studies.*—Tritiated thymidine ([$^3$H]-TdR; Radiochemical Centre, Amersham; sp. act. 21 Ci/mmol) was diluted for injection with 0·9% NaCl. [$^3$H]-TdR was given i.p. to the rats in the following doses: animals to be killed for the percentage labelled mitoses (PLM) and 1h (flash) labelling were given a single injection of 2 $\mu$Ci/g body weight in 1 ml 0·9% NaCl; animals for repeated tumour-labelling experiments received 0·5 $\mu$Ci/g [$^3$H]-TdR every 6 h and a further 0·5 $\mu$Ci/g 1 h before killing. Autoradiographs of 4$\mu$m paraffin-embedded hemisections of the tumours were prepared using the dipping technique (Baserga & Malamud, 1969) with Ilford K-2 emulsion (Ilford Ltd., Ilford, Essex). After 14 days exposure, the autoradiographs were processed with D-19 developer and F-5 fixer (Rogers, 1967) and stained with haematoxylin and eosin. For the PLM curves, 500 anaphases and metaphases were counted "blind", and for the flash and repeated labelling indices (% labelled cells) a total of 2,000 cells was scored.

To determine background counts in autoradiographs, a tumour-bearing animal was injected with [$^3$H]-TdR and the tumour fixed 30 min later. This period is too brief for mitoses to become labelled (Lala, 1971). Therefore, any silver grains over such mitoses in an autoradiograph are due to background radiation. Sections of the tumour were prepared in large numbers and used as standards for processing with each batch of autoradiographs. Background in the majority of autoradiographs averaged less than 1 grain per cell and if this amount of labelling was exceeded the preparations were not used. Cells with 5 or more grains per cell were considered labelled.

Vincristine (Oncovin, Eli Lilly, Basingstoke) was injected i.p. into the rats at a dose of 1 mg/kg in 1 ml 0·9% NaCl. Paraffin hemisections (4 $\mu$m) of the tumours were stained as above and the mitotic index (% mitoses) determined by counting 4,000 cells.

FIG. 1.—Growth curve of the Yoshida sarcoma (■) and tumour-volume changes after curative hyperthermia (intra-tumour temperature 42°C for 1 h) on Day 9 after implantation (1·0–1·5 ml, ●) and on Day 16 (3·0–3·5 ml, ▲). Figures in brackets indicate the numbers of tumours (rats) used to construct the curves. Vertical bars indicate s.d.

During heating of the 1·0–1·5ml tumours, intra-abdominal temperature of the rats remained within the normal range (36·8–39·5°C); with the 3·0–3·5ml tumours, body temperature usually increased to 39·5–40·5°C during hyperthermia.

### RESULTS

### *Cell-population kinetics of the untreated tumour*

The volume-growth curve of the Yoshida sarcoma is shown in Fig. 1. There were 3 distinct phases of growth in untreated tumours: (i) a lag phase until Day 5 after implantation, (ii) a phase of rapid exponential growth between Days 5 and 10 when the tumour volume increased from 0·15 to 2·0 ml with a doubling time ($T_d$) of 36 h, (iii) a slower exponential increase between Days 10 and 20 as tumour volume increased from 2·0 to 5·0 ml, with a $T_d$ of 144 h. At volumes above 5·0 ml, the tumour was no longer confined to the foot, and tumour volume could not be accurately determined. Cell-kinetic studies were carried out at two different tumour volumes:

(a) 1·0–1·5 ml (rapid exponential growth).
(b) 3·0–3·5 ml (slower exponential growth).

FIG. 2.—Percentage labelled mitosis (PLM) curves of the Yoshida sarcoma at volumes of (a) 1·0–1·5 ml, (b) 3·0–3·5 ml.

TABLE I.—*Median cell-cycle times of the Yoshida sarcoma*

| Tumour volume (ml) | Median cell-cycle time (h) | $T_{G2}$ | $T_S$ | $T_{G1}$ | $T_M$ |
|---|---|---|---|---|---|
| 1·0–1·5 | 14·1 | 4·0 | 9·7 | — | 0·4 |
| 3·0–3·5 | 13·8 | 3·2 | 10·2 | — | 0·6 |

Median cell-cycle and phase times were obtained from the PLM curves of Fig. 2 by inspection at the 50% points. $T_M$ was determined from the metaphase-arrest curves of Fig. 3, using the equation:

$$T_M = \frac{I_M}{K_b}$$

(Lala, 1971) where $I_M$ = percentage mitoses in tumours from untreated animals, and $K_b$ = birth rate of cells (rate of entry into mitosis).

FIG. 3.—Mitotic accumulation after vincristine arrest in unheated Yoshida sarcoma at volumes of (a) 1·0–1·5 ml, (b) 3·0–3·5 ml. Cell birth rate in (a) calculated from the slope of the percentage mitoses line, as per Wright *et al.* (1972) (9·4/2·0 = 4·7%/h). Unlabelled broken lines indicate 95% confidence limits.

*PLM curves*

The PLM curves of the smaller and larger Yoshida sarcoma are shown in Fig. 2. The $T_c$ was similar at both tumour volumes, being 14·1 h in the 1·0–1·5 ml tumour and 13·8 h in the 3·0–3·5 ml tumour (Table 1). No $G_1$ period was detected at either tumour volume.

*Labelling index, birth rate and computed kinetic parameters*

The labelling index (LI) at 1 h de-

TABLE II.—*Cell kinetic parameters*

| Tumour volume (ml) | Doubling time, $T_d$ (h) | $K_b$ ($h^{-1}$) | LI | Growth fraction (GF) | Cell-loss factor (Ø) |
|---|---|---|---|---|---|
| 1·0–1·5 | 36 | 0·047 | 52·5 | 67·8 | 59·0 |
| 3·0–3·5 | 144 | 0·021 | 28·2 | 39·6 | 75·9 |

Growth fraction was determined by the cell-cycle stage technique (Lala, 1971) using the following equation:

$$GF = \frac{LI}{\left\{\exp\left(T_S \frac{\ln 2}{T_C}\right) - 1\right\} \exp\left(T_G + T_M \frac{\ln 2}{T_C}\right)}$$

Cell loss factor, Ø (Steel, 1968) was determined as follows:

$$Ø = \frac{K_L}{K_b}$$

where

$K_L$ = rate of cell loss from the population $= \frac{\ln(1+GF)}{T_c} - \frac{\ln 2}{T_d}$

$K_b$ = birth rate, measured as the rate of entry into mitosis using vincristine for mitotic arrest (Aherne *et al.*, 1977).

creased with increasing tumour volume from 52·5% in the 1·0–1·5 ml tumour to 28·2% in the 3·0–3·5 ml tumour. The birth rate measured by vincristine blockade (Fig. 3) decreased from 4·7 to 2·1% in the larger tumour (Table II).

The slower growth of the larger tumour compared with the 1·0–1·5 ml tumour appeared to be due to a decrease in growth fraction (from 67·8 to 39·6%) and an increase in cell-loss factor from 59 to 75·9% (Table II).

### *Effect of hyperthermia on cell-kinetic parameters*

*Growth curve.*—The effect of 1 h at 42°C on tumour volume is shown in Fig. 1. Treatment of the tumour at 1·0–1·5 ml caused a decrease in tumour volume and complete regression within 14 days. There was a 96% cure rate of the animals. Treatment of the tumour at 3·0–3·5 ml produced a restraint in growth followed by partial regression, although variation in response between tumours was considerable (Fig. 1). No tumour increased in volume after heat, however. Hyperthermia in rats bearing these larger tumours constituted a hazard to the host, leading to a significantly reduced lifespan of 27·4 ± 5·5 days compared to 44·6 ± 7·23 days in untreated tumour-bearing controls ($P < 0·001$). At autopsy, animals that died at 27 days

FIG. 4.—[³H]-TdR labelling in 1·0–1·5 ml Yoshida sarcoma after hyperthermia. (a) Flash (1 h) labelling index in tumours after curative heating. (b) Repeated labelling in unheated Yoshida tumours (△), tumours labelled 0–48 h after hyperthermia (●), tumours in which labelling was terminated 14 h after heating (□), or tumours in which labelling began at 14 h and continued to 48 h after heating (○).

showed enhanced spread of tumour locally and to distant sites; the role of heat in this enhanced dissemination has been discussed previously (Dickson, 1976).

*The* 1·0–1·5 *ml tumour*

The effect of heating at 42°C on the [$^3$H]-TdR LI is illustrated in Fig. 4a. Immediately after hyperthermia, LI was depressed from a control level of 52·5% to 5% labelled cells. Labelling remained inhibited and showed considerable variation between tumours in the period from 0 to 24 h, but recovered to near control levels by 48 h after heating. From ~50% at 48 h, the LI declined to 35% at 72 h, 10% at 96 h and zero by 5 days after heat as the tumour regressed.

Changes in tumour-cell kinetics in the 48 h after heat were investigated in more detail by repeated [$^3$H]-TdR labelling (Fig. 4b). In controls LI increased to a plateau of almost 80% labelled cells after 8 h. After hyperthermia, LI was depressed to 5% labelled cells immediately after heat, and then recovered rapidly to a maximum of 60% after 8 h. An equally rapid decline in LI to a nadir of 5% at 14 h then preceded an erratic recovery to a plateau of ~60% labelled cells 36–48 h after hyperthermia. When repeated labelling was carried out from 0 to 14 h after heat and then ceased, the increase in LI from 14 to 48 h was small. When, however, labelling was commenced at 14 h after heat and continued to 48 h, LI recovered to the 60% region by 30 h.

The effect of 1 h at 42°C on entry of tumour cells into mitosis is shown in Fig. 5. Immediately after heat, the rate of entry into mitosis was lower and more variable than in controls (Figs. 5, 3a). The mitotic rate had partially recovered by 6 h after heat. At this time, tumours had a high base-line mitotic index (5% compared with 2·3% in controls) indicating that some cells were blocked in mitosis by the heat. Entry into mitosis at 12 and 18 h was almost completely inhibited. The mitotic rate began to increase again at 24 h, although variation between tumours was such that no meaningful value for $K_b$ could be computed. By 48 h after heat, entry into mitosis had reached control levels, and variability between tu-

FIG. 5.—Mitotic accumulation in the 1·0–1·5 ml Yoshida sarcoma after vincristine arrest at different times after hyperthermia. Vincristine was injected at each time indicated (0–96 h) and mitotic accumulation studied over the following 2·5 h. The least-squares best-fit line ±s.d. is plotted. $K_b$ is the cell birth rate as % cells/h, calculated as in Fig. 3a. After the 12, 18 and 24h intervals, the increase in mitotic index was not significant at the 5% level, so $K_b$ was indistinguishable from zero.

FIG. 6.—PLM curve of the 1·0–1·5ml Yoshida sarcoma 30–54 h after hyperthermia (○ - - - ○) constructed using data from 15 tumours. The PLM curve of the untreated tumour (●——●) is included for comparison.

FIG. 7.—[$^3$H]-TdR labelling in the 3·0–3·5ml Yoshida sarcoma after hyperthermia. (a) Flash-labelling index in tumours after curative heating. The normal range represents the mean LI (28·2 ± 4·6% labelled cells) calculated from 10 control tumours. (b) Repeated labelling in control tumours (△ - - - △) and in tumours after heating for 1 h at 42°C (●——●).

mours was similar to that in controls (Figs 5, 3a). From 48 h onwards, the rate of entry into mitosis declined as the tumour regressed. Mitoses were increasingly restricted to the periphery of the tumour, which by 96 h consisted of a central core of necrotic tissue surrounded by a thin rim of viable cells. Tumours appeared totally necrotic by 6 days after heat.

The PLM curve of the tumour 30 h after heat is shown in Fig. 6. The $T_c$ of 13 h derived from the curve was not significantly different from the 14·1 h in untreated controls.

*The* 3·0–3·5 *ml tumour*

The effect of 1 h at 42°C on the [$^3$H]-TdR LI is shown in Fig. 7a. Immediately after heating, no labelled cells were seen in the tumour. The LI slowly increased from 1·0% at 12 h to 2·5% at 24 h until it reached a maximum of 16% labelled cells at 48 h. There was then a progressive decrease to a mean of 8% labelled cells at 72 h and to zero 7 days after heating. Tumours 7–8 days after hyperthermia were completely necrotic and contained no [$^3$H]-TdR labelled cells or mitoses. The effect of hyperthermia on repeated [$^3$H]-TdR labelling in the tumour is shown in Fig. 7b. In untreated controls, the LI increased from 28% at 1 h to a plateau of ~55% by 18–24 h. Following hyperthermia, the LI varied between 6 and 8% from 0 to 24 h. After 24 h, a slow increase occurred to 32% at 48 h.

### DISCUSSION

The decreased growth rate of the Yoshida sarcoma at the larger volume ($T_d$ 144 h at 3·5 ml compared to 36 h at 1·5 ml) resulted from a decreased growth fraction and an increase in cell-loss factor; the cell-cycle time of the tumour was unaltered (Tables). A similar finding has been reported previously in solid tumours, in which $T_c$ appears to be a relatively constant factor and alterations in growth rate are due mainly to changes in GF and Ø (Watson, 1976; Aherne *et al.*, 1977; Steel, 1977). The median $T_c$ of the Yoshida sarcoma remained a stable parameter in cells repopulating the tumour after hyperthermia (Fig. 6); the changes produced by curative heating concerned cell loss and an alteration in the relationship between the P and Q cell compartments (*vide infra*) rather than cell-generation times.

Alterations in the [$^3$H]-TdR flash LI indicated a complex sequence of events in the tumour cell population after heating (Fig. 4a). The decreased LI immediately after hyperthermia reflected killing of cells in S phase and/or a 2–3 h block in the progression of cells into and through S phase. After release of the block, the partially synchronized cells appeared to enter S phase and cause a maximum in LI at 8 h (Fig. 4b). The decline in LI to a minimum at 14 h must be a consequence of further cell death. Thus, more than 90% of the cells proliferating after hyperthermia had been lost by 14 h, the median $T_c$ of the tumour. This would imply that cells damaged at the time of heating had progressed through one cell cycle and died, possibly in mitosis. It seems unlikely that the rapid recovery of labelling from 14 to 36 h was caused by cells in cycle at the time of heat treatment. A more likely explanation is the entry into S phase of a population of cells not proliferating at the time of heat treatment; this would repopulate the tumour from 14 h onwards. Evidence in favour of this hypothesis (Fig. 4b) is that when repeated labelling was terminated 14 h after heating (the time of maximum cell death) there was only a small increase in subsequent labelling. When repeated labelling was commenced at 14 h, LI increased to near control values. These two experiments support the hypothesis that the recovery in proliferation from 14 h after heat was mainly due to recruitment of cells from the Q compartment of the tumour population. A similar effect was noted by Lücke-Hühle & Dertinger (1977) using V79 spheroids *in vitro*. Cells in the centre of the spheroids had a low proportion of P cells under normal conditions. Heating at 42°C for 4 h caused extensive loss from the population of cells with a high GF at the periphery of the spheroids, and appeared to stimulate the entry of Q cells in the centre of the spheroids into cycle. This caused an increase in the proportion of cells in S phase at the interior of the spheroids for 12–24 h after heating. Preferential destruction of P cells followed by recruitment of Q cells into cycle has been reported after radiation or chemotherapy to solid tumours, and the timing of recovery has been used to plan fractionated therapy regimens (see Steel (1977) for refs.).

The stathmokinetic study detailed in Fig. 5 gave supportive evidence for the inferences drawn from Fig. 4. The blockade of entry into mitosis immediately after heat paralleled the inhibition of entry into S phase at this time. This blocking effect of heat on progression round the cell cycle has previously been noted *in vitro* by Rao & Engelberg (1965) and Sisken *et al.* (1965) and more recently by Palzer & Heidelberger (1973*a*) Kase & Hahn (1975) Gerweck & Dewey (1976) Lücke-Hühle & Dertinger (1977) and Sapareto *et al.* (1978). The release of the mitotic block in the Yoshida sarcoma and entry into mitosis at 6 h (Fig. 5) again paralleled the entry of cells into S phase at this time (Fig. 4a). The high base-line mitotic index of 5% at 6 h (compared to 2·3% in controls) could indicate cells blocked in mitosis, possibly in the early stages of mitotic death. The decline in mitotic rate to almost 0 cells/h at 12 h and 18 h (Fig. 5) is consistent with destruction of the P cell population from 8–14 h after heating, as inferred from the repeated-labelling study. Recovery of the mitotic rate by 48 h (Fig. 5) supports the inference drawn from the

[$^3$H]-TdR studies (Figs 4a, b) that proliferation in the Yoshida sarcoma had regained control values by this time after heating. The progressive decline in mitotic rate after 48 h (Fig. 5) to zero at 6 days as the tumour regressed, followed a similar time course to the decline in flash LI (Fig. 4a). By Day 9 the tumour was totally necrotic, and mitoses and [$^3$H]-TdR labelled cells were no longer seen in sections of such tumours. It is not clear to what extent the reduction in LI and mitotic rate from 2–9 days was due to inhibition of cell division and wastage by cell loss or to delayed cell killing due to a cytotoxic effect of heat. The mitotic rate and LI of cells in viable areas of the tumour 72 and 96 h after heat (Figs 4a, 5) were considerably less than in controls, so a reduced rate of cell production was at least partially implicated in the destruction of the tumour-cell population 2–9 days after heat.

A similar pattern in cytokinetic response was seen in the 3·0–3·5 ml tumour after heating to that found in the 1·0–1·5 ml tumour (Figs 4a, 7a). However, in the 48 h immediately after hyperthermia, there were differences in response between the tumours, indicating that cells in the larger tumour may have suffered more damage. In the 3·0–3·5 ml tumours, labelling did not increase significantly until 48 h after hyperthermia, and there was no peak in LI at 6–8 h as in the 1·0–1·5 ml tumour (Figs 4b, 7b). It is apparent that the larger tumour with a smaller growth fraction (39·6% *vs* 67·8%, Table II) and a larger fraction of Q cells, was at least as heat-sensitive as the smaller tumour. Thus, it would seem that only a fraction of the Q-cell population in the larger tumour was able to enter the cell cycle and repopulate the tumour. The Q-cell compartment of tumours is thought to be heterogeneous (Sarna, 1974; Gelfant, 1977) and may contain cells of differing heat sensitivity. Untreated 3·0–3·5 ml tumours contained large necrotic zones, probably bordered by Q cells remote from the tumour micro-circulation. Such cells would be deficient in $O_2$ and nutrients, conditions which have been shown to sensitize cells to heat *in vitro* (Gerweck *et al.*, 1974; Bass *et al.*, 1978). The results imply that there is no simple relationship between the proliferative status and the thermosensitivity of tumour-cell populations *in vivo*.

In both tumour sizes, failure of cells surviving 48 h after heat treatment to maintain the growth of the tumour may be due to 3 mechanisms:

(1) Failure to repair sublethal damage followed by delayed, heat-induced cell killing. The finding of proliferating cells in the tumour up to 6 days after curative heating confirms *in vitro* findings that several mitoses (up to 10 in Yoshida sarcoma) may occur before the expression of lethal hyperthermic damage (Palzer & Heidelberger, 1973*a*). The role of repair processes in hyperthermic cell damage has been discussed by Bronk (1976).

(2) Preferential eradication of clonogenic cells in the tumour and cell population decline due to cell loss.

(3) The operation of host factors in the destruction of the tumour. It has been demonstrated that regression of the Guerin carcinoma in the rat (Szmigielski & Janiak, 1978) and the VX2 carcinoma in the rabbit (Shah & Dickson, 1978) after local hyperthermia, are accompanied by stimulation of a host anti-tumour immune response. A recent review (Dickson, 1978) indicates that in both inbred and outbred animals (and also in man) immunogenic tumours are more readily cured by heat that non-immunogenic tumours, and it has been reported that cure of the immunogenic MC7 sarcoma in rats and the non-immunogenic VX2 carcinoma in rabbits may be abrogated by immunosuppression of the host (Shah & Dickson, 1979). Little definitive information is available on the immunogenicity of the Yoshida sarcoma, although immune factors seem to be involved in cure of the tumour by chemotherapy (Fox & Gregory, 1972). In rats with 1·0–1·5 ml Yoshida sarcomas, metastatic tumour cells are present in the regional lymph nodes. Cure of such animals by heating the primary tumour for 1 h at 42°C, and subsequent resistance of the hosts to tumour inoculation, implies the generation of an anti-tumour response by hyperthermia (Dickson & Ellis, 1976). The two distinct exponential phases in the growth curve of the Yoshida sarcoma (Fig. 1) could be interpreted as the operation of anti-tumour immunity from 10 days after implantation. However, in tumours grown in the thigh muscles or s.c. in the flank, growth retardation did not occur until tumour volumes of 8–10 ml were attained, and the growth pattern was more Gompertz-like, with a smooth decrease in growth from an initial exponential phase. It is believed, therefore, that the volume curve of the tumour reflects the anatomical characteristics and functional restrictions of the foot as a site for growth rather than a host anti-tumour response.

We thank Mrs J. Hogg for expert technical assistance. The work was supported by the North East of England Council of the Cancer Research Campaign.

### REFERENCES

AHERNE, W. A., CAMPLEJOHN, R. S. & WRIGHT, N. A. (1977) *An Introduction to Cell Population Kinetics*. London: Arnold.

BASERGA, R. & MALAMUD, D. (1969) *Autoradiography*. New York: Harper & Row.

BASS, H., MOORE, L. J. & COAKLEY, W. T. (1978) Lethality in mammalian cells due to hyperthermia under oxic and hypoxic conditions. In *Cancer Therapy by Hyperthermia and Radiation*. Baltimore: Urban & Schwarzenberg. p. 172.

BHUYAN, B. K., DAY, K. J., EDGERTON, C. E. & OGUNBASE, O. (1977) Sensitivity of different cell lines and of different phases in the cell cycle to hyperthermia. *Cancer Res.*, **37**, 3780.

BRONK, B. V. (1976) Thermal potentiation of mammalian cell killing: clues for understanding and potential for tumour therapy. *Adv. Radiat. Biol.*, **6**, 267.

DICKSON, J. A. (1976) Hazards and potentiators of hyperthermia. In *Proc. Int. Symp. Cancer Treatment by Hyperthermia and Radiation*. Baltimore: *Am. Coll. Radiol. Press*. p. 134.

DICKSON, J. A. (1977) The effects of hyperthermia in animal tumour systems. In *Selective Heat Sensitivity of Cancer Cells. Recent Results Cancer Res.*, **59**, 43.

DICKSON, J. A. (1978) Sensitivity of human cancer to hyperthermia. In *Proc. Conf. Clin. Prospects for Hypoxic Cell Sensitizers and Hyperthermia.* (Eds) W. L. Caldwell and R. E. Durand. Madison: Univ. Wisconsin Press. p. 174.

DICKSON, J. A. & CALDERWOOD, S. K. (1976) *In vivo* hyperthermia of Yoshida tumour induces entry of non-proliferating cells into cycle. *Nature*, **263**, 772.

DICKSON, J. A. & ELLIS, H. A. (1974) Stimulation of tumour cell dissemination by raised temperature (42°C) in rats with transplanted Yoshida tumours. *Nature*, **248**, 354.

DICKSON, J. A. & ELLIS, H. A. (1976) The influence of tumour volume and the degree of heating on the response of the solid Yoshida sarcoma to hyperthermia (40–42°C). *Cancer Res.*, **36**, 1188.

DICKSON, J. A. & SUZANGAR, M. (1974) *In vitro–in vivo* studies of the susceptibility of the solid Yoshida sarcoma to drugs and hyperthermia (42°C). *Cancer Res.*, **34**, 1263.

FOX, B. W. & GREGORY, C. J. (1972) A study of the immunosuppressive activity of methylene dimethane sulphonate (MDMS) in relation to its effectiveness as an anti-tumour agent. *Br. J. Cancer*, **26**, 84.

GELFANT, S. (1977) A new concept of tissue and tumor cell proliferation. *Cancer Res.*, **37**, 3845.

GERWECK, L. E. & DEWEY, W. C. (1976) Variation in response to heat during the mammalian cell cycle. In *Proc. Int. Symp. Cancer Treatment by Hyperthermia and Radiation*. Baltimore: Am. Coll. Radiol. Press. p. 16.

GERWECK, L. E., GILLETTE, E. L. & DEWEY, W. C. (1974) Killing of Chinese hamster cells *in vitro* by heating under hypoxic or aerobic conditions. *Eur. J. Cancer*, **10**, 691.

HILL, B. T. (1978) The management of human "solid" tumours: some observations on the irrelevance of traditional cell cycle kinetics and the value of certain recent concepts. *Cell. Biol. Int. Rep.*, **2**, 215.

HILL, B. T. & BASERGA, R. (1975) The cell cycle and its significance for cancer treatment. *Cancer Treat. Rev.*, **2**, 159.

KASE, K. & HAHN, G. M. (1975) Differential heat response of normal and transformed human cells in tissue culture. *Nature*, **255**, 228.

LALA, P. K. (1971) Studies on tumour cell population kinetics. *Methods Cancer Res.*, **6**, 3.

LÜCKE-HÜHLE, C. & DERTINGER, H. (1977) Kinetic response of an *in vitro* "tumour-model" (V-79 spheroids) to 42°C hyperthermia. *Eur. J. Cancer*, **13**, 23.

PALZER, R. J. & HEIDELBERGER, C. (1973*a*) Studies on the quantitative biology of hyperthermic killing of HeLa cells. *Cancer Res.*, **33**, 415.

PALZER, R. J. & HEIDELBERGER, C. (1973*b*) Influence of drugs and synchrony on the hyperthermic killing of HeLa cells. *Cancer Res.*, **33**, 422.

RAO, P. N. & ENGELBERG, J. (1965) HeLa cells: effects of temperature on the life cycle. *Science*, **148**, 1092.

ROGERS, A. W. (1967) *Techniques of Autoradiography*. Amsterdam: Elsevier. p. 253.

ROSSI-FANELLI, A., CAVALIERE, R., MONDOVI, B. & MORICCA, G. (1977) (Eds) *Recent Results Cancer Res.*, **59**.

SAPARETO, S. A., HOPWOOD, L. E., DEWEY, W. C., RAJU, M. R. & GRAY, J. W. (1978) Effects of hyperthermia on survival and progression of Chinese hamster ovary cells. *Cancer Res.*, **38**, 393.

SARNA, G. (1974) The resting cell: a chemotherapeutic problem. *Biomedicine*, **20**, 322.

SHAH, S. A. & DICKSON, J. A. (1978) The effect of hyperthermia on the immunocompetence of VX2 tumor-bearing rabbits. *Cancer Res.*, **38**, 3523.

SHAH, S. A. & DICKSON, J. A. (1979) Effect of hyperthermia on the phagocytic activity of tumour bearing animals. *Br. J. Cancer*, **40**, 818.

SISKEN, J. E., MORASCA, L. & KIBBY, S. (1965) Effects of temperature on the kinetics of the mitotic cycle of mammalian cells in culture. *Exp. Cell Res.*, **39**, 103.

STEEL, G. G. (1968) Cell loss from experimental tumours. *Cell Tissue Kinet.*, **1**, 193.

STEEL, G. G. (1977) *Growth Kinetics of Tumours.* Oxford: Clarendon Press. p. 268.

STEWART, H. L., SNELL, K. C., DUNHAM, L. J. & SCHLYEN, S. M. (1959) *Transplantable and transmissible tumors of animals.* Washington, D.C.: Armed Forces Inst. Pathol. p. 352.

STREFFER, C. (1978) (Ed.) *Cancer Therapy by Hyperthermia and Radiation.* Baltimore: Urban & Schwarzenberg.

SZMIGIELSKI, S. & JANIAK, M. (1978) Reaction of cell-mediated immunity to local hyperthermia of tumors and its potentiation by immunostimulation. In *Cancer Therapy by Hyperthermia and Radiation.* Baltimore: Urban & Schwarzenberg. p. 80.

THRALL, D. E., GERWECK, L. E., GILLETTE, E. L. & DEWEY, W. C. (1976) Response of cells *in vitro* and tissues *in vivo* to hyperthermia and X-irradiation. *Adv. Radiat. Biol.*, **6**, 211.

WATSON, J. V. (1976) The cell proliferation kinetics of the EMT6/M/AC mouse tumour at four volumes during unperturbed growth *in vivo*. *Cell Tissue Kinet.*, **9**, 147.

WESTRA, A. & DEWEY, W. C. (1971) Variation in sensitivity to heat shock during the cell cycle of Chinese hamster cells *in vitro*. *Int. J. Radiat. Biol.*, **19**, 467.

WIZENBERG, M. J. & ROBINSON, J. E. (1976) (Eds) *Proc. Int. Symp. Cancer Therapy by Hyperthermia and Radiation.* Baltimore: Amer. Coll. Radiol. Press.

WRIGHT, N., MORLEY, A. & APPLETON, D. (1972) Variation in the duration of mitosis in the crypts of Lieberkuhn of the rat; A cytokinetic study using vincristine. *Cell Tissue Kinet.*, **5**, 351.

YOSHIDA, T. (1971) Comparative studies of ascites hepatoma. *Methods Cancer Res.*, **6**, 97.